# Lack of Evidence That Neural Empathic Responses Are Blunted in Excessive Users of Violent Video Games: An fMRI Study

**DOI:** 10.3389/fpsyg.2017.00174

**Published:** 2017-03-08

**Authors:** Gregor R. Szycik, Bahram Mohammadi, Thomas F. Münte, Bert T. te Wildt

**Affiliations:** ^1^Department of Psychiatry, Social Psychiatry and Psychotherapy, Hannover Medical SchoolHanover, Germany; ^2^Department of Neurology, University of LübeckLübeck, Germany; ^3^Clinical Neuroscience Lab, International Neuroscience InstituteHanover, Germany; ^4^Institute of Psychology II, University of LübeckLübeck, Germany; ^5^Department of Psychosomatic Medicine and Psychotherapy, LWL University Hospitals of the Ruhr-UniversityBochum, Germany

**Keywords:** video games, violence, desensitization, General Aggression Model, Catalyst Model

## Abstract

The use of violent video games has been often linked to increase of aggressive behavior. According to the General Aggression Model, one of the central mechanisms for this aggressiveness inducing impact is an emotional desensitization process resulting from long lasting repeated violent game playing. This desensitization should evidence itself in a lack of empathy. Recent research has focused primarily on acute, short term impact of violent media use but only little is known about long term effects. In this study 15 excessive users of violent games and control subjects matched for age and education viewed pictures depicting emotional and neutral situations with and without social interaction while fMRI activations were obtained. While the typical pattern of activations for empathy and theory of mind networks was seen, both groups showed no differences in brain responses. We interpret our results as evidence against the desensitization hypothesis and suggest that the impact of violent media on emotional processing may be rather acute and short-lived.

## Introduction

The possible influence of violent video games (VVG) on human aggressive behaviour is hotly debated. According to research done in the context of the General Aggression Model (GAM) a direct and causal relationship between the use of VVG and aggressiveness (Anderson and Bushman, 2002) is postulated: aggressive or impulsive behavior is a short term result of both personal and situational variables like exposure to VVG. Therefore, an increase of aggression after exposure to VVG is hypothesized to appear as a result of cognitive cuing effects (Anderson and Dill, 2000). Long-term exposure to VVG on aggressive behavior according to the GAM leads to an increase in aggressive personality traits by learning, rehearsal and reinforcement of aggression-related knowledge structures. Also, a desensitization against violent content and a decrease of empathy and prosocial behavior has been postulated (Sparks and Sparks, 2002; Anderson et al., 2003; Huesmann et al., 2003).

The alternative Catalyst Model postulates only little or no effects of VVG use on human aggressive behavior (Ferguson et al., 2008). Following this model, aggressive behavior results primarily from biological factors and VVG only shape the style of aggressive expression. This view criticizes the GAM because of the discrepancy between its predictions and the recent violence statistics (Ferguson, 2010), small effect sizes or poor quality meta-analyses (Kutner and Olson, 2008; Ferguson, 2015a), and the publication bias or selective reporting of only significant data (Elson and Ferguson, 2014; Ferguson, 2015b).

The literature regarding short term desensitization effects of VVG has been inconsistent. Some short term desensitization effects could be shown regarding physiological reactivity, i.e., reduction in heart rate or galvanic skin reactions to violent stimuli (Carnagey et al., 2007a,b; Staude-Muller et al., 2008). The P300 component of the event-related potential has also been found to be reduced after VVG use (Bartholow et al., 2006; Engelhardt et al., 2011). Other studies using violent and non-violent versions of the same game could not find differences in physiological markers like heart rate or skin conductance (Ballard et al., 2012; Chittaro and Sioni, 2012). A recent meta-analysis suggests a relationship between VVG use and decrease in empathy (and more desensitization) but only for short term effects (Anderson et al., 2010). Long term effects were not analyzed in this meta-analysis because of a lack of pertinent studies. To our knowledge only two studies focussed on long term desensitization effects of VVG use by the means of fMRI. In the first study small effects were shown but the results were not adequately corrected for multiple comparisons (Montag et al., 2012) which raise the possibility that the reported differences would not have survived adequate correction. Our group studied 28 male excessive VVG users and a matched control group with two experiments using emotional picture stimuli from the IAPS data base (Szycik et al., 2016). The user group had at least 3 h of VVG abstinence prior to experiment making the design more suitable for analyzing long term effects. VVG users showed similar brain responses as control subjects for emotional material indicating no specific desensitization effect of excessive long lasting VVG use.

As Montag et al. (2012) and Szycik et al. (2016) the current study focused on the long-term desensitization effects of VVG use but this time with an emphasis on empathy. For this reason we used a picture set, patterned after the materials of the Adult Attachment Projective Picture System (AAP; George and West, 2001), designed to elicit empathic / emotional reactions in situations with and without social interaction (Krämer et al., 2010; Beyer et al., 2014a,b). According to the Perception-Action-Model (PAM) of empathy (Preston and de Waal, 2002) and in keeping with previous results using the same stimulus set we hypothesized that pictures depicting another person’s emotional state should elicit empathic reactions and concomitant neural activations. Furthermore, according to the desensitization hypothesis derived from the GAM that excessive VVG users should show decreased brain activity in the empathy network (Bzdok et al., 2012).

## Materials and Methods

All subjects gave written informed consent in accordance with the Declaration of Helsinki. The protocol was approved by the ethic committee of the Medical School Hannover. All participants were also provided with short briefing prior to the experiment. First possible contradictions for MRI measurements were cleared by standard questionnaire and then all necessary information regarding the study was given (e.g., experimental task). The subjects had also the possibility to ask questions before the experiment. After the experiment all subjects were provided with standard debriefing containing additional information regarding the study.

### Participants

As use of VVG and aggressive behavior is more prevalent in men, only male participants were recruited. Inclusion criterion for the VVG user group was consumption of violent games of the first-person shooter category (e.g., Counterstrike, Call of Duty or Battlefield) for at least 4 years and for at least 2 h daily. First-person shooter games are centered on combat situations seen from the first-person perspective, involving virtual weapons (mostly automatic rifles). Control subjects did not have any experience with VVG (self-report). We also excluded all control subjects that reported a daily use of any video games. All participants were free of psychiatric and neurological disorders as assessed clinically by the senior author, a board-certified psychiatrist. One participant of the VVG group was excluded from the analysis due to taking antidepressant medication. All of the subjects had normal or corrected to normal vision. Experimental and control groups were matched for school education and age.

For the experiment 15 VVG users (mean age 22.8 ± 4.3 years) and 15 control subjects (mean age 22.1 ± 3.0 years) were recruited (difference n.s.). The VVG users had played violent games since 13.1 ± 4.4 years for about 4.0 ± 1.3 h daily.

To avoid possible immediate effects of violent games all participants refrained from playing for at least 3 h prior to the experiment during which time they were informed about the experiment and were prepared for data acquisition. The actual time without playing VVG before the experiment was considerably longer in most subjects.

### Stimuli and Design

**FIGURE 1:**
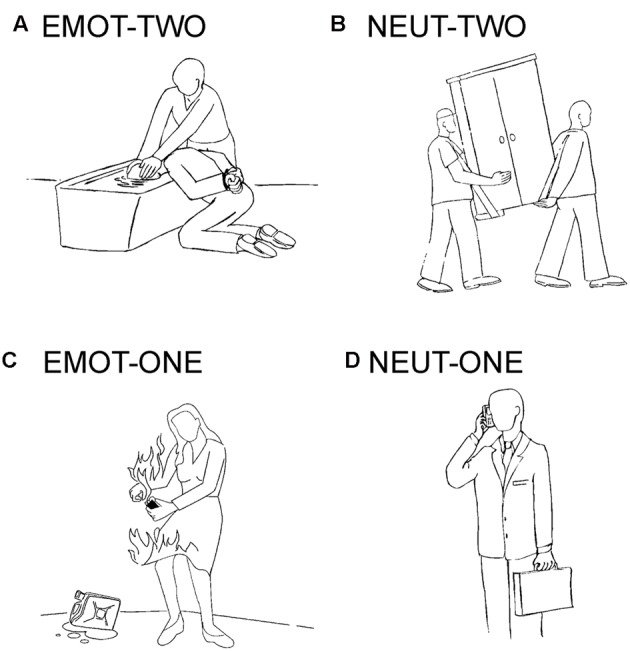
**Example stimuli.** Depicted are examples of the drawings used in this study: **(A)** emotionally negative social situations involving two interaction partners (EMOT-TWO), **(B)** emotionally neutral social situations also involving two interaction partners (NEUT-TWO), **(C)** emotionally negative situations involving one person (EMOT-ONE), and **(D)** emotionally neutral situations involving one person (NEUT-ONE).

The experimental stimulation in this study is based on a previous publication and uses black- line drawings on a gray background (Krämer et al., 2010; Beyer et al., 2014a,b). The drawings were assigned to four conditions: emotionally negative social situations involving two interaction partners (EMOT-TWO), emotionally neutral social situations also involving two interaction partners (NEUT-TWO), emotionally negative situations involving one person (EMOT-ONE), and emotionally neutral situations involving one person (NEUT-ONE). Negative emotional stimuli depicted emotions like anger, sadness, pain or anxiety (**Figure 1**). The stimuli had been previously rated for their emotional content with consistent results across the individual subjects and significant differences for the specific categories (Krämer et al., 2010).

The stimuli were presented for 4 s in pseudorandom order each with varying interstimulus interval (ISI). For each of the four experimental categories 24 different stimuli were used. Between the stimuli a black central fixation cross was presented on gray background. ISI varied pseudo-randomly within each category with 15 intervals of 6 s duration, 5 intervals of 8 s duration and 2 intervals each of 10 s and 12 s duration. During fMRI scanning participants were instructed to watch the pictures carefully and imagine how they would feel in the depicted situation. Presentation software (Neurobehavioral Systems, Inc.) was used to deliver stimuli. Stimuli were presented via an MRI-compatible video display mounted into prepared glasses (CinemaVision, Resonance Technology Inc., USA). Prior to fMRI scanning a test picture was presented to ensure good visibility of stimuli for each participant.

### Image Acquisition

Magnetic-resonance images were acquired on a 3-T Siemens Magnetom Scanner (Erlangen, Germany) equipped with a standard head coil. A total of 545 T_2_^∗^-weighted volumes of the whole brain (EPI-sequence; TR 2000 ms, TE 30 ms, flip angle 80∘, FOV 192 mm, matrix 64^2^, 34 slices, slice thickness 3 mm, interslice gap 0.75 mm) near to standard bicommisural (AC-PC) orientation were collected. After the functional measurement a 3D high resolution T_1_-weighted volume for anatomical information (MPRAGE-sequence; matrix 192 × 256^2^, 1 mm isovoxel) was recorded. The subject’s head was fixed during the entire measurement to avoid head movements.

### Acquisition of Psychometric Data

Prior to fMRI scanning data from different psychological questionnaires was collected. We used the German adaptation (“Der Saarbrücker Persönlichkeitsfragebogen zur Messung von Empathie”) of the Interpersonal Reactivity Index (IRI) with its four subscales: PT- perspective-taking, FS- fantasy scale, EC- empathic concern, and PD- personal distress (Davis, 1980). To analyze potential group differences in aggressiveness we collected the data from the short version of the German questionnaire for aggressiveness factors K-FAF: “Kurzfragebogen zur Erfassung von Aggressivitätsfaktoren” (Heubrock and Petermann, 2008). To analyze the ability in emotional understanding, processing, or description the German adaptation of the Toronto Alexithymia Scale (Bagby et al., 1994) was used. To assess relevant personality traits we used the German adaptation of the Temperament and Character Inventory TCI (Cloninger, 1994) and The Inventory of Clinical Personality Accentuations (“Inventar Klinischer Persönlichkeitsakzentuierungen”) to screen clinical personality aspects (Andresen, 2006).

### fMRI Data Analysis

Analysis and visualization of the data were performed using Brain Voyager QX (Brain Innovation BV, Maastricht, The Netherlands) software (Goebel et al., 2006). First, a correction for the temporal offset between the slices acquired in one scan was applied. For this purpose the data was cubic spline interpolated. After this slice scan time correction a 3D motion correction was performed by realignment of the entire measured volume set to the first volume by means of trilinear interpolation. Thereafter, linear trends were removed and a high pass filter was applied resulting in filtering out signals occurring less than 2 cycles in the whole time course. Structural and functional data were spatially transformed into the Talairach standard space (Talairach et al., 1988) using a 12-parameter affine transformation. Functional EPI volumes were spatially smoothed with an 8 mm full-width half-maximum isotropic Gaussian kernel to accommodate residual anatomical differences across volunteers.

For the statistical model a design matrix including all conditions of interest was specified using a hemodynamic response function. This function was created by convolving the rectangle function with the model of Boynton et al. (1996) using Δ = 2.5, τ = 1.25 and *n* = 3. Thereafter, a multi-subject random effects (RFX) analysis of variance model (ANOVA) with two main within-subject factors and one between-subject factor was used for identification of significant differences in hemodynamic responses. The first within subject factor was emotional content (EMOT vs. NEUT), the second within subject factor was social relation (TWO vs. ONE). The between-subject factor was group (VVG users vs. control subjects). Additional as regressors of no interest we used overall six translation and rotation vectors derived for each dataset during the 3D motion correction.

Main effects of all factors and their interaction were considered. The false discovery rate threshold of q(FDR) < 0.01 (Genovese et al., 2002) was chosen for identification of the activated voxels. Voxels fulfilling these criteria are reported. The centers of mass of suprathreshold regions were localized using Talairach coordinates and the Talairach Daemon tool (Lancaster et al., 2000).

## Results

### Questionnaire Data

Group differences were obtained only for the factor Novelty Seeking of the Temperament and Character Inventory [*t*(28) = 2.126, *p* < 0.042] and the scale Antisocial Personality of The Inventory of Clinical Personality Accentuations [*t*(28) = 3.255, *p* < 0.003]. VVG users showed higher scores for Novelty Seeking (*M* = 25.27, *SD* = 4.59 vs. *M* = 20.47, *SD* = 7.44) and for Antisocial Personality (*M* = 22.13, *SD* = 5.89 vs. *M* = 16.13, *SD* = 4.03) in comparison to controls. No further group differences were apparent in the questionnaires, in particular no differences were seen for empathy and aggression measures (for the overview of all questionnaire data see Supplementary Material).

### fMRI

**FIGURE 2:**
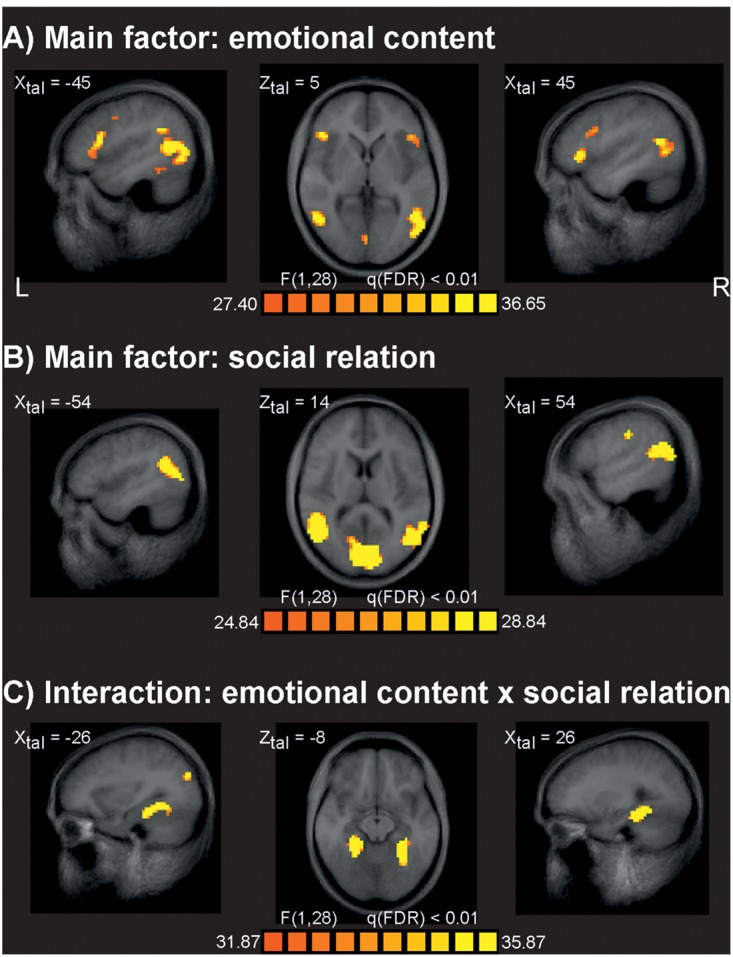
**Results of the fMRI data analysis.** **(A)** Brain sites identified for the main factor emotional content responding for stimuli with emotional negative and neutral valence. **(B)** Brain sites identified for the main factor social relation, responding for stimuli depicting one person or two persons in social interaction. **(C)** Brain sites identified for the interaction of the main factor emotional content and social interaction. The factor group revealed no significant brain sites, also all other interactions resulted in no significant results. L, leftt; R, right; XZtal, Talairach coordinates.

The analysis of fMRI data revealed at q(FDR) < 0.01 strong effects for the main factor emotional content (**Figure 2A**). At a less strict level of q(FDR) < 0.05 additional typical activations often seen for the processing of emotionally relevant material were found, such as bilateral limbic structures including both amygdalae. The main effect of social interaction was significant for several brain areas at q(FDR) < 0.01 (**Figure 2B**) similar to earlier studies (Krämer et al., 2010; Beyer et al., 2014a,b). There were no significant differences between VVG users and controls at the level of q(FDR) < 0.01. To check for weak effects, this analysis was repeated at a very liberal threshold of *p* = 0.01, uncorrected, but again no reliable activation differences between groups were seen. We also analyzed all possible interaction effects at the q(FDR) < 0.01 significance level. Only the interaction of emotional content by social relation resulted in reliable brain activations related primarily to bilateral parahippocampal gyrus (**Figure 2C**). All other interactions including the factor group were not significant at this level. **Table 1** gives an overview about the identified brain sites for the main factors and interaction of the ANOVA analysis.

**Table 1:** Brain areas identified for the ANOVA.

Brain structure	Hemisphere	Talairach center of mass			Cluster size (mm^3^)
			
		*x*	*y*	*z*	
**Main Factor: emotional content**					
*Inferior Frontal Gyrus, BA13*	R	44	26	3	1242
*Inferior Frontal Gyrus, BA9*	R	41	10	28	1458
Superior Temporal Gyrus, BA39	R	50	-52	14	4401
*Lingual Gyrus, BA18*	R	6	-75	3	648
Medial Frontal Gyrus, BA9	L	-1	47	30	918
*Precentral Gyrus, BA44*	L	-42	18	8	3645
Middle Frontal Gyrus, BA6	L	-39	-2	44	945
*Middle Temporal Gyrus, BA39*	L	-48	-57	9	9180
**Main Factor: social relation**					
*Postcentral Gyrus, BA2*	R	58	-19	33	837
*Superior Temporal Gyrus, BA22*	R	48	-53	16	6480
Cuneus, BA18	R/L	1	-72	18	34911
*Superior Temporal Gyrus, BA39*	L	-48	-59	17	6750
**Interaction: emotional content x social relation**					
*Parahippocampal Gyrus, BA36*	R	24	-38	-10	3294
*Parahippocampal Gyrus, BA37*	L	-28	-44	-10	3861
Middle Temporal Gyrus, BA19	L	-34	-78	26	540


## Discussion

A central claim of the GAM regarding the effects of VVG is desensitization toward emotional stimuli. Although some evidence has been provided for short term effects of VVG in the sense of a decreased empathy and increased of aggressiveness (Carnagey et al., 2007a,b; Staude-Muller et al., 2008), long term effects have not been intensively investigated. Long term effects were the focus of the present study, which assessed neural responses to stimuli designed to elicit empathic reactions. To rule out short term effects of VVG, users had ben abstinent for at least 3 h prior to the measurements. Contrary to our initial hypothesis of a reduced activity in empathy related brain regions in VVG users, the fMRI data did not provide evidence for a neural desensitization in the processing emotionally salient stimuli. In fact, the responses of both groups were very similar and no group differences were observed even at relaxed statistical thresholds. This lack of a group main effect and of interaction effects involving the group factor is not due to a general lack of emotional reactivity in our participants. Indeed, we found robust activations for the factor emotional content in our dataset (**Figure 2A**) similar to those found previously in studies using the same materials (Krämer et al., 2010; Beyer et al., 2014a,b). These activations included areas already known as involved in processing of emotional content (limbic structures, ventromedial and ventrolateral prefrontal cortex) and areas involved in mentalizing or theory-of-mind process (e.g., regions around superior temporal sulcus) (Bzdok et al., 2012, 2013; Mutschler et al., 2013; Mitchell and Phillips, 2015; Morelli et al., 2015). Our paradigm clearly is sensitive to differences in emotional content. Also, aspects of social relation could be reliable observed in our data and were in line with the previous research (Adolphs, 2003; Krämer et al., 2010; Beyer et al., 2014a,b).

Thus, the lack of group differences in our fMRI data dues not suggests, that excessive VVG use leads to long term emotional desensitization and a blunting of neural responses related to empathy. This is corroborated by the questionnaire data which did not reveal differences between VVG users and controls for empathy and aggression measures, even though some differences emerged for measures assessing novelty seeking and antisocial personality.

Most previous studies have focused on immediate effects of VVG use (Brockmyer, 2015). For example, Weber et al. (2006) looked at fMRI activations during the performance of violent computer games and reported a suppression of amygdala and anterior cingulate gyrus activity which was taken to suggest a blunted emotional reactivity. Other researchers reported a decreased interaction between amygdala and the lateral orbitofrontal cortex directly after exposure to violent media (Kelly et al., 2007). Gentile et al. (2014) reported suppression of fMRI responses to violent compared to non-violent video games in VVG users. Evidently, these studies used stimulation with violent media/games immediately before or even during the experiment and therefore the results may by influenced not only by desensitization but also by other factors such as increased attention toward motor actions or immediate activation of aggressive cognitions. In any case, these reflect only possible short term influence of VVG on emotional processing. Studies focussing on long term effects are rare and show results that are in line with the present study (Szycik et al., 2016).

The missing group effect in our fMRI data is not really surprising giving the fact that both groups showed also no differences in empathy and aggressiveness as assessed by psychological tests. Our data therefore is in line with the Catalyst Model of violent media influence on individual behavior which posits that these do not increase aggressive behavior but may influence the way how aggressive behavior is displayed. Therefore, aggressiveness itself results more from other aspects than violent media use. This idea is supported by our data. VVG users differ in personality trait Novelty Seeking. As Novelty Seeking is highly correlated with Sensation Seeking (Zuckerman et al., 1993) subjects with high values on this scale are vulnerable for risky activities and tend to excessive behavior resulting often both in substance related and behavioral addictions like e.g., excessive use of video games (Bardo et al., 1996; McCoul and Haslam, 2001; Roberti, 2004; Grucza et al., 2006; Wang et al., 2015). VVG users of our study also showed high values on the antisocial scale of the clinical personality inventory. This again may be the basis for specific problematic behavior often suggested for this population. In this sense VVG use might be a yet another symptom not the cause of problems in this group. One interesting question arises from the fact that the significant group difference in antisocial personality found in this study was not accompanied by significant differences in empathy scores. Empathy is only one part of many (e.g., disregard for social norms, rules, and obligations, incapacity to maintain enduring relationships, incapacity to experience guilt or to profit from experience) involved in the psychological construct of antisocial personality. Keeping that in mind or VVG group could score significant higher on antisocial personality without differing from the control group in empathy scores.

Before concluding our results we want to put some attention to the limitations of the study. Thus we did not found group differences in our fMRI data set. Null findings in imaging studies are notoriously problematic (Hupe, 2015) and may result also from to small effects in relation to the extent of the population included. One possibility to handle this problem is to decrease statistical threshold used with the risk of making “false positive” conclusions. We tried to maximize our ability to find group differences and to safeguard against “false negatives” and lowered the statistical threshold used to very liberal one of *p* = 0.01, uncorrected. But also after the adaptation of the threshold no group differences could be found. Second relevant limitation of this study relies on the fact, that our both populations were not controlled for consumption of other than VVG violent media, e.g., violent cinema movies or internet content. Thus we cannot rule out the possibility that our control subjects consumed in similar excessive extent other violent contents and have experienced desensitization at same level as our experimental group.

To summarize, our results provide additional evidence against the desensitization hypothesis of VVG use and human aggression. Research on media impact on aggressive behavior should focus on short term (influencing the subject’s state) as well as long term impact (possibly influencing trait aggressiveness). Moreover, additional paradigms should be employed, e.g., facial expression tasks (van Zutphen et al., 2015), to examine these aspects within VVG users. Also the use of more ecological valid paradigms could be promising to put more light on this topic. Interesting approach could be to put individuals with VVG use and controls into situations requiring acting upon emotional stimuli like it is the case in the Taylor Aggression Paradigm. A number of recent imaging studies have used a version of this paradigm to assess impulsive aggression in response to provocation (Krämer et al., 2007; Beyer et al., 2014a,b, 2015; Dambacher et al., 2015; Gan et al., 2015).

## Author Contributions

GS designed the study, collected and analyzed the data, interpreted the results and wrote the manuscript. BM collected the data and supported the interpretation of the results. TM supported the interpretation of the data and wrote the manuscript. BtW designed the study, collected the data and supported the interpretation of the results.

## Conflict of Interest Statement

The authors declare that the research was conducted in the absence of any commercial or financial relationships that could be construed as a potential conflict of interest.
