# Supplementary material for: Lack of Evidence That Neural Empathic Responses Are Blunted in Excessive Users of Violent Video Games: An fMRI Study
Source: Front Psychol. 2017 Mar 8;8:174. doi: 10.3389/fpsyg.2017.00174 (PMC5341328; doi:10.3389/fpsyg.2017.00174)
Supplement: Supplementary file 1 [file Table_1.docx]

|  | Supplemental data: Questionnaire data | |  |  |
| --- | --- | --- | --- | --- |
|  |  |  |  |  |
|  | Questionnaire/Scala | VVG group | Control group |  |
|  |  | M (SD) | M (SD) |  |
| Interpersonal Reactivity Index | |  |  |  |
|  | *Perspective Taking* | 13.5 (2.2) | 14.6 (2.9) | T=1.19, n.s. |
|  | *Fantasy* | 12.9 (3.8) | 13.2 (2.0) | T=0.24, n.s. |
|  | Empathic Concern | 13.7 (2.7) | 14.0 (2.1) | T=0.38, n.s. |
|  | *Personal Distress* | 9.9 (3.4) | 9.6 (2.3) | T=0.25, n.s. |
| K-FAF | |  |  |  |
|  | *Self-aggression* | 17.6 (10.1) | 13.4 (8.6) | T=1.22, n.s. |
|  | Aggression-inhibition | 14.7 (3.6) | 15.6 (5.7) | T=0.50, n.s. |
|  | Aggression total score | 68.9 (20.2) | 56.6 (24.3) | T=1.51, n.s. |
| Toronto Alexithymia Scale | |  |  |  |
|  | *Difficulty Describing Feelings* | 13.6 (4.5) | 14.2 (4.2) | T=0.38, n.s. |
|  | *Difficulty Identifying Feeling* | 12.5 (3.8) | 14.0 (5.6) | T=0.86, n.s. |
|  | *Externally-Oriented Thinking* | 13.5 (2.8) | 13.7 (3.7) | T=0.22, n.s. |
| TCI |  |  |  |  |
|  | Novelty seeking | 25.3 (4.6) | 20.5 (7.4) | T=2.13, p<0.042 |
|  | *Harm avoidance* | 12.5 (8.2) | 15.0 (5.5) | T=1.00, n.s. |
|  | *Reward dependence* | 16.1 (3.7) | 16.5 (4.0) | T=0.33, n.s. |
|  | *Persistence* | 3.7 (2.7) | 4.7 (2.1) | T=0.91, n.s. |
|  | *Self-directedness* | 28.3 (9.7) | 30.9 (5.6) | T=0.90, n.s. |
|  | Cooperativeness | 26.7 (7.8) | 30.2 (4.9) | T=1.49, n.s. |
|  | Self-transcendence | 12.2 (6.3) | 10.3 (6.3) | T=0.81, n.s. |
| IKP |  |  |  |  |
|  | Paranoid personality disorder | 24.5 (4.6) | 21.5 (4.1) | T=1.92, n.s. |
|  | Schizoid personality disorder | 17.7 (5.2) | 14.7 (5.8) | T=1.38, n.s. |
|  | Schizotypal personality disorder | 17.7 (4.3) | 15.3 (5.1) | T=1.24, n.s. |
|  | Antisocial personality disorder | 22.1 (5.9) | 16.1 (4.0) | T=3.25, p<0.003 |
|  | Borderline personality disorder | 17.3 (5.2) | 15.9 (4.1) | T=0.82, n.s. |
|  | Impulsive-explosive personality disorder | 18.3 (5.6) | 19.1 (7.7) | T=0.32, n.s. |
|  | Histrionic personality disorder | 20.4 (5.6) | 21.8 (3.9) | T=0.79, n.s. |
|  | Narcissistic personality disorder | 21.1 (6.1) | 21.7 (6.1) | T=0.27, n.s. |
|  | *Avoidant personality disorder* | 20.4 (9.0) | 17.6 (5.7) | T=1.02, n.s. |
|  | *Dependent personality disorder* | 21.2 (5.4) | 18.8 (5.2) | T=1.23, n.s. |
|  | Obsessive-compulsive personality disorder | 21.8 (5.2) | 21.3 (5.1) | T=0.28, n.s. |
